# Comparison of textural properties and structure of gels prepared from cooked rice grain under different conditions

**DOI:** 10.1002/fsn3.916

**Published:** 2019-01-28

**Authors:** Tomoko Sasaki, Junko Matsuki, Koichi Yoza, Junichi Sugiyama, Hideo Maeda, Akiko Shigemune, Ken Tokuyasu

**Affiliations:** ^1^ Food Research Institute National Agriculture and Food Research Organization Tsukuba Ibaraki Japan; ^2^ Kagoshima‐Osumi Food Technology Development Center Kanoya, Kagoshima Japan; ^3^ Central Region Agricultural Research Center National Agriculture and Food Research Organization Joetsu, Niigata Japan; ^4^ Western Region Agricultural Research Center National Agriculture and Food Research Organization Fukuyama, Hiroshima Japan

**Keywords:** apparent amylose content, chain length distribution, gel network, high‐speed shear homogenization, rice gel, textural properties

## Abstract

The objective of this study was to investigate the effects of rice variety, water content, and preparation temperature on the textural properties of gels processed from cooked rice grains via high‐speed shear homogenization. Rice gels were prepared from seven high‐amylose rice varieties. The results demonstrated the significant differences in rice gel hardness and hardening rates during storage based on the rice variety used. The proportion of short chains of amylopectin was negatively correlated with the hardness of the rice gel. The sample temperature before shear treatment also influenced the rice gel hardness. Rice gels prepared from cooked rice maintained at 75°C prior to homogenization showed a higher breaking force than those from cooked rice at 25°C. Observation using scanning electron microscopy demonstrated the tendency of the cooked rice sample maintained at 75°C to form a finer gel network after homogenization than those at 25°C from the same rice variety.

## 1 INTRODUCTION

Gels derived from various cereals are prominent in solid foods. The rheological properties of rice flour gel contribute to the texture and palatability of rice flour products such as noodles. Starch is the primary component of rice and an important ingredient in processed foods made from rice. When starch granules are heated in excess water, the granules swell and gelatinize, during which process the molecular order of the starch granule is destroyed. When cooling a sufficiently concentrated suspension of gelatinized starch, starch retrogradation occurs, resulting in gel formation. Retrograded starch is common in the human diet, because it is formed by cooking or food processing. The physical properties of starch, such as gelatinization, retrogradation, and gelation, play an important role in determining the quality of rice products. Starches consist of amylose and amylopectin. The short‐term development of retrogradation in starch gels is attributed to the gelation of the solubilized amylose fraction (Miles, Morris, & Ring, 1985), and the long‐term changes in starch granules are attributed to the recrystallization of the amylopectin fraction (Abd Karim, Norziah, & Seow, 2000). The quality of rice noodles, one of the primary rice gel products, is greatly dependent on the amylose content of rice (Cham & Suwannaporn, 2010).

Rice is a staple food in Asian countries, and various rice flour products have been developed. Rice gels, including rice noodles, are typically made from rice flour. However, Shibata et al. (2012) demonstrated that high‐amylose rice gel, which was prepared from cooked rice grains using high‐speed shear treatment, showed unique viscoelastic properties, and it may have the potential to modify the texture of rice products. Kokawa et al. (2017) also revealed the possibility of utilizing these rice gels in bread making and the potential to significantly modify the product texture by changing the conditions of rice gel preparation. Rice flour gels are made from rice flour dispersion, and the effects of the processing conditions on their rheological properties have been reported in previous studies (Jena & Bhattacharya, 2003; Kapri & Bhattacharya, 2008; Xu, Xiong, Li, & Zhao, 2008). However, the effect of varying the processing conditions during the production of rice gels from cooked rice grains is not well understood because of new material. The present study aimed to elucidate the influence of high‐amylose rice cultivar, water content, and preparation temperature on the textural properties of gels processed from cooked rice grains using high‐speed shear homogenization. In addition, the relationship between the starch characteristics of rice and its textural properties was examined.

## 2 MATERIALS AND METHODS

### 2.1 Rice samples

Seven high‐amylose rice varieties were used to prepare rice gels. HA1 and HA2 were grown at the Central Region Agricultural Research Center, NARO (Niigata, Japan), and HA3 and HA4 were grown at the Western Region Agricultural Research Center, NARO (Hiroshima, Japan). Yumetoiro (Yume), Momiroman (Momi), and Hoshiyutaka (Hosh) were obtained from farmers.

### 2.2 Chemical analysis

The total starch content of the rice was determined using an assay kit (Megazyme International Ireland, Ireland). Apparent amylose content was determined by iodine colorimetry (Juliano et al., 1981). The chain length distribution of amylopectin in starches isolated from the seven rice varieties tested was analyzed by high‐performance anion‐exchange chromatography on a CarboPac PA‐1 column equipped with a pulsed amperometric detector (HPAEC‐PAD, Dionex, USA). The sample solution was prepared using the method described by Nagamine and Komae (1996). After isoamylase debranching, the sample solution was eluted at 1 ml/min with a linear gradient of 50–1000 mM sodium acetate in 100 mM NaOH. Amylopectin branch chain types were classified as follows: DP6‐12 (A chain), DP13‐24 (B1 chain), and DP25‐36 (B2 chain) (Hanashiro, Abe, & Hizukuri, 1996). Protein content (*N* × 5.95) was determined using the Kjeldahl method.

### 2.3 Preparation of gels from cooked rice grains

In the present study, three types of cooked rice were prepared. Rice samples were weighed in a 100‐ml homogenizer cup, and 2× (33%, w/w), 3× (25%, w/w), or 4× (20%, w/w) the mass in distilled water was added to bring the total mass to 80 g (these preparations are denoted 2× water, 3× water, and 4× water, respectively). The rice was cooked in a water bath at 98°C for 30 min with an aluminum foil cover, and the cooked rice samples were maintained in a water bath at 25°C or 75°C for 30 min immediately after cooking and then homogenized using an Excel auto homogenizer (Nihonseiki Kaisha Ltd., Japan) at 5000 rpm for 3 min. Stainless steel petri dishes measuring 40 mm in diameter and 15 mm in height were filled with the homogenized rice pastes and sealed in airtight bags, followed by cooling at 5°C for 1 h, 1 day, 3 days, or 5 days.

### 2.4 Solubilized starch measurement

Solubilized starch of rice paste was quantified using a method modified from that described by Han and Hamaker (2000). The homogenized rice paste (3.0 g) prepared from cooked rice grains was weighed into 50‐ml screw‐capped tubes, and 30 ml of distilled water (60°C) was added. The tubes were placed in a shaking water bath at 60°C for 10 min and cooled in cold water (10°C) for 5 min. They were centrifuged at 3370 g for 15 min. The supernatant was transferred to a new tube, and an amylose/amylopectin assay kit (Megazyme International Ireland) was used to determine the total starch and amylose contents in the recovered supernatant. The percent soluble starch, amylose, and amylopectin contents were corrected for the original dry weight of the rice.

### 2.5 Scanning electron microscopy

Cross sections of rice gels were observed using a scanning electron microscopy (SEM) (JSM‐7001F, JEOL, Japan). Freeze‐dried gel samples were fractured and mounted on a specimen holder and then coated with osmium and platinum before SEM observation. The obtained specimens were examined at an accelerating voltage of 5 kV.

### 2.6 Textural analysis

Texture measurements of cooked rice grains and rice gels were performed using a texture analyzer (TA‐XT Plus, Stable Micro Systems, UK) equipped with a 5‐kg load cell. For texture measurements on cooked rice grains, a compression test was performed using a cylindrical probe (35 mm diameter). After the cooked rice was maintained at 25°C or 75°C for 30 min, a single cooked rice grain was placed in the center of a base plate and uniaxially compressed by a probe at a constant rate of deformation (0.5 mm/s) to 90% of its original thickness. The forces required for 25% and 90% strains were used to compare surface hardness and overall hardness, respectively (Okadome, Toyoshima, & Ohtsubo, 1999). For the measurement of rice gels stored for 1 h, 1 day, 3 days, and 5 days, the compression test was performed using a cylindrical probe with a diameter of 10 mm. Gels remained in the petri dishes in which they were prepared for testing. The center of a gel was uniaxially compressed by the probe at a constant rate of deformation of 1 mm/s to 95% of the original gel thickness.

### 2.7 Statistical analysis

Chemical analysis was performed in duplicate or greater. Textural analysis of rice gels was performed in triplicate or greater. For the measurement of the grain texture of cooked rice, the mean of 30 single cooked rice grains was calculated. A general linear model (SAS Institute, USA) was used to analyze the data. Analysis of variance and Tukey's studentized test were conducted at a 5% significance level.

## 3 RESULTS AND DISCUSSION

### 3.1 Chemical analysis

Calculated protein, total starch, and apparent amylose contents, as well as the chain length distribution of amylopectin, are shown in Table 1. The protein content and total starch content of the rice samples were 6.3%–9.1% and 86.0%–91.2%, respectively. Of the seven rice varieties tested, Yume showed the highest protein content and the lowest total starch content. The seven high‐amylose rice varieties tested had a wide range of apparent amylose content (19.0%–27.9%). The peak area ratios of DP 6‐12, DP 13‐24, and DP 25‐36 were 17.8%–32.5%, 46.1%–52.7%, and 8.7%–14.7%, respectively. HA2 contained the lowest portions of the short chains (DP 6‐12) and highest portions of DP 25‐36, whereas Hosh contained the highest portions of the short chains and lowest portions of DP 25‐36. In particular, differences in the percentages of short amylopectin chains (DP 6‐12) were pronounced between each variety.

**Table 1:** Protein, total starch, apparent amylose contents, and chain length distribution of amylopectin

Sample	Protein content (%, db)	Total starch content (%, db)	Apparent amylose content (%)	Chain length distribution of amylopectin (%)		
				DP6‐12	DP13‐24	DP25‐36
HA1	7.4	90.8	27.5	24.9	52.7	10.1
HA2	7.9	86.6	22.9	17.8	46.9	14.7
HA3	6.7	90.9	24.5	31.4	46.1	9.6
HA4	7.1	90.7	19.0	30.2	47.1	9.9
Yume	9.1	86.0	25.7	30.3	48.7	11.0
Momi	6.3	91.2	22.7	31.8	46.8	10.2
Hosh	6.9	88.1	27.9	32.5	46.8	8.7

### 3.2 Solubilized starch

The solubilized starch contents of pastes prepared from the seven cooked rice samples were 0.4%–19.7% (2× water), 0.8%–29.6% (3× water), and 1.0%–32.3% (4× water) (Table 2). The solubilized amylose contents of the paste were 0.2%–17.2% (2× water), 0.3–28.0% (3× water), and 0.3%–21.8% (4× water); thus, the amount of water added to cook the rice influenced the solubilized starch content. Table 2 shows that cooking the rice in 2× water resulted in considerably lower solubilized starch content, and cooking in 3× and 4× water showed similar values. Comparing the solubilized starches of the different rice varieties, HA2 and HA4 showed significantly higher solubilized starch and amylose contents than the other varieties. Solubilized starch and amylose contents were negatively correlated with apparent amylose content in rice, and results indicated that more amylose leached out of homogenized pastes prepared from cooked rice with lower amylose content. Rice varieties other than HA2 and HA4 showed higher solubilized amylopectin content than solubilized amylose, suggesting that the amylose in HA2 and HA4 was more susceptible to leaching in hot water. The temperature of cooked rice samples before homogenizing had some influence on the content of solubilized starch. For rice cooked in 3× water, the solubilized starch and amylose contents of pastes prepared from cooked rice maintained at 25°C were higher than those prepared at 75°C. This influence was not observed for rice cooked in 2× and 4× water.

**Table 2:** Solubilized starch for the paste prepared from cooked rice^a^ ^,^ ^b^

Sample	Solubilized starch (%)			Solubilized amylose (%)			Solubilized amylopectin (%)		
	×2 water	×3 water	×4 water	×2 water	×3 water	×4 water	×2 water	×3 water	×4 water
25°C									
HA1	2.8c	16.2c	15.2de	0.3d	6.3d	4.4d	2.5b	9.9a	10.8a‐c
HA2	13.4b	22.6b	16.9c	7.3c	14.5bc	5.4c	6.2a	8.1a‐d	11.5a
HA3	1.7c	12.6d	15.6cd	0.2d	3.6de	4.4de	1.5bc	9.0ab	11.3ab
HA4	19.7a	29.6a	27.9b	17.2a	28.0a	17.5b	2.5b	1.6fg	10.3bc
Yume	2.3c	3.6g	2.7h	0.4d	0.7e	0.7h	1.8b	2.9fg	2.0g
Momi	1.9c	11.1de	14.6de	0.2d	2.6de	3.4e‐g	1.7b	8.6a‐c	11.2ab
Hosh	2.5c	9.2ef	11.0f	0.4d	2.2e	2.4g	2.1b	7.0b‐d	8.5e
75°C									
HA1	3.1c	6.9f	8.7g	0.4d	1.2e	2.9fg	2.7b	5.6de	5.8f
HA2	11.9b	21.4b	28.1b	6.0c	12.9c	17.6b	5.9a	8.5a‐c	10.5a‐c
HA3	1.9c	4.1g	11.4f	0.2d	0.7e	2.6g	1.6bc	3.3ef	8.8de
HA4	18.0a	27.9a	32.3a	13.0b	18.1b	21.8a	5.0a	9.8a	10.5bc
Yume	0.4c	0.8h	1.0h	0.2d	0.3e	0.3h	0.2c	0.5g	0.7h
Momi	3.0c	7.1f	11.4f	0.3d	1.1e	2.4g	2.7b	6.0d	9.0de
Hosh	1.7c	8.3f	13.5e	0.2d	1.7e	3.7d‐f	1.5bc	6.7cd	9.8cd

^a^In the same column, values with the same letter are not significantly different at *p* < 0.05. ^b^Dry weight percentage of original rice.

### 3.3 Microstructure of gels prepared from cooked rice

**Figure 1:**
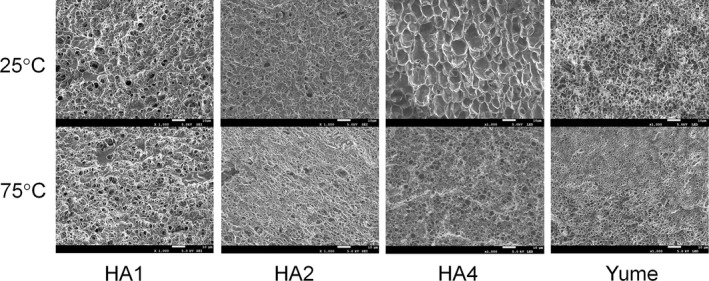
Scanning electronic micrographs of rice gels on day 5

Figure 1 shows the microstructures of gels prepared from four representative varieties under SEM, which revealed different structures when maintained at 25°C and 75°C after cooking. The gel prepared from cooked rice maintained at 75°C prior to homogenization formed a more fibrous and denser structure, and the gel from cooked rice maintained at 25°C formed a more cellular structure, particularly for HA4. Kokawa et al. (2017) prepared two types of rice gel, namely, hot rice gel and cooled rice gel, and demonstrated that the hot rice gels contained more bubbles and had a smaller mean bubble diameter compared to cooled rice gel. In the present study, a similar tendency was observed, suggesting that the cooked rice grain maintained at a higher temperature (75°C) formed a finer gel network following homogenization than that maintained at a lower temperature (25°C).

### 3.4 Textural properties of cooked rice and rice gels

**Figure 2:**
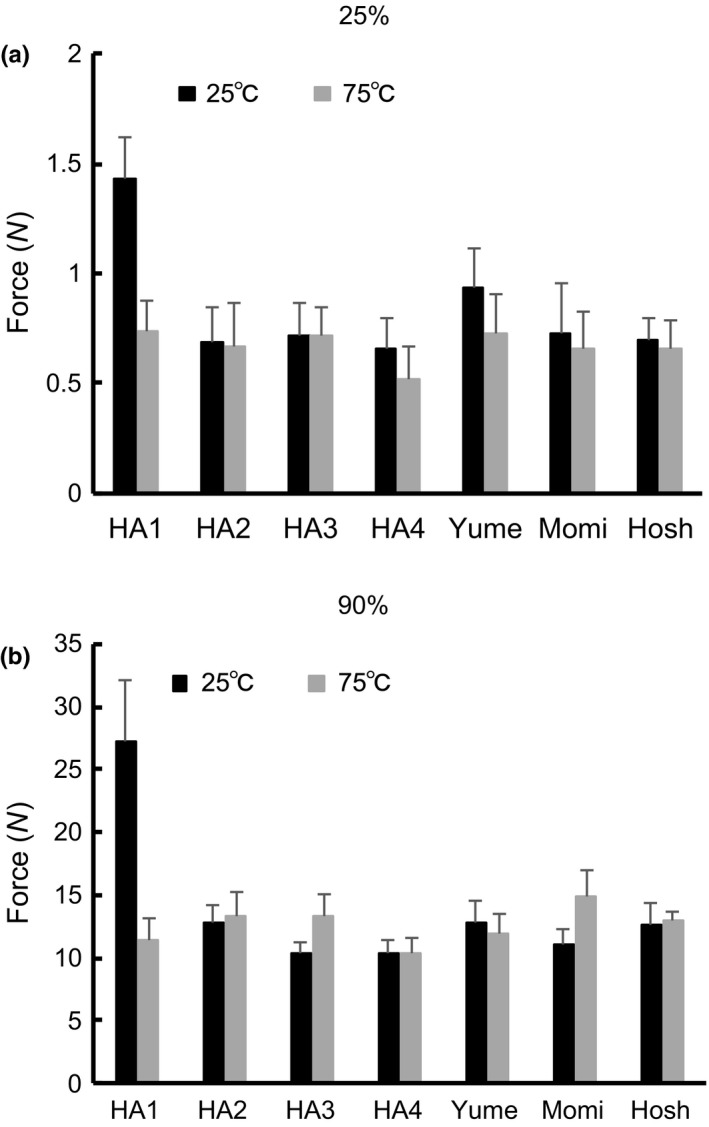
Compressive force required to yield 25% and 90% strains from the cooked rice grains

Figure 2 compares the compressive forces required on cooked rice grains maintained 25°C and 75°C to achieve 25% and 90% strains. The compressive forces required for 25% and 90% strains were used to compare surface hardness and overall hardness, respectively. The surface and overall hardness of cooked rice grains varied between rice varieties and temperature conditions. Of all rice varieties tested, HA1 showed a markedly higher compressive force at 25% and 90% strains when maintained at 25°C than the other varieties, and the difference in compressive force was minimal at 75°C. At 25% strain, HA1 and Yume showed a significant difference between grains maintained at 25°C versus 75°C. At 90% strain, HA1, HA3, and Momi showed a significant difference between grains maintained at 25°C and 75°C. HA4 showed the lowest compressive force under any conditions. Relationships between compressive force and chemical contents were evaluated using the compressive force and the chemical analysis data. The compressive force at 25% and 90% strains for cooked rice maintained at 25°C was highly correlated with the peak area ratio of DP13‐24 (*r* = 0.98, 0.95, *p* < 0.01). The apparent amylose content was significantly correlated with the compressive force at 25% strain for cooked rice maintained at 75°C (*r* = 0.78, *p* < 0.05). The influences of starch characteristics on textural properties of cooked rice grains have been reported elsewhere. Li, Prakash, Nicholson, Fitzgerald, and Gilbert (2016) demonstrated that the difference in amylose molecular size contributed to the difference in textural properties of cooked rice grains as well as amylose content and amylopectin structure. No significant relationship between compressive force and protein content of rice was observed.

**Figure 3:**
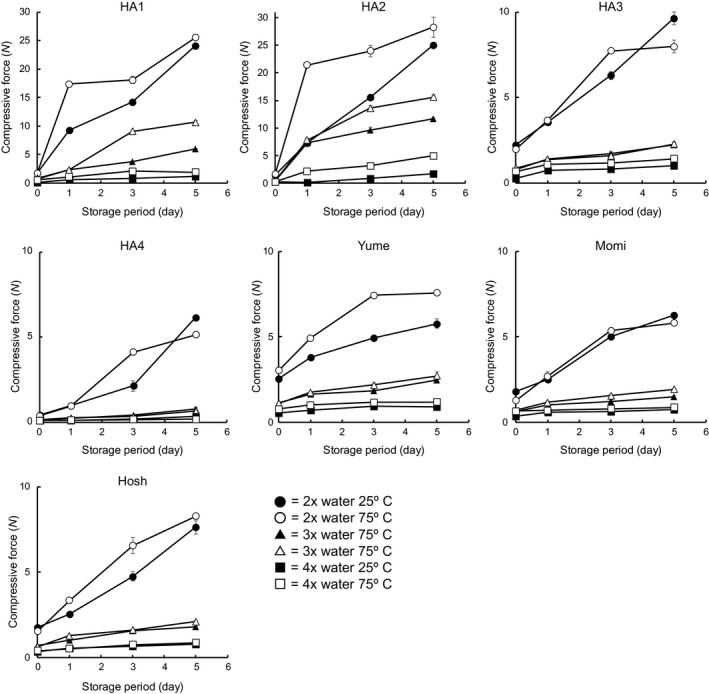
Comparison of compressive forces required to yield 20% strain from the rice gels prepared from seven rice varieties

Figure 3 shows a comparison of the changes in compressive force during storage of the prepared rice gels. The force required to achieve 20% strain before fracture was used as an indicator of gel hardness for all samples, because no fracture point was found for a part of samples. HA1 and HA2 showed a sharp increase in hardness at an early stage of storage and notably increasing force throughout the storage time, especially when prepared with 2× water. HA4 exhibited the lowest force throughout the storage time under any conditions, although this variety showed a distinct drop in hardness between the preparations with 2× water and 3× water. On the other hand, HA3, Yume, Momi, and Hosh showed intermediate gel hardness characteristics. The gel hardness or hardening rate of all rice varieties showed a dependence on concentration, and the strength of this dependence differed between varieties. The water content of rice gels markedly affected their hardening rate. For HA3, HA4, Yume, Momi, and Hosh, the hardening rate was significantly increased by increasing the gel concentration from 25% (3× water) to 33% (2× water). HA2 showed a remarkable increase in the hardening rate when the gel concentration was increased from 20% (4× water) to 25% (3× water). The starch concentration has been previously reported to influence the mechanism of starch retrogradation; Keetles, van Vliet, and Walstra (1996a,b) indicated that the swelling area of individual starch granules is restricted in the concentrated system, resulting in tighter granule packing. However, when the starch concentration is low, the swollen granules cannot fully occupy the available area, and amylose easily leaches out during heating, forming an amylose gel layer after cooling (Flipse, Keetles, Jacobson, & Visser, 1996). The influences of rice gel water content on the hardening rate of gels were found to differ between rice varieties. Because starch is a main component of rice, this implies that the influences of starch concentration on the rate of starch retrogradation also differs between rice varieties, and the differences in starch characteristics between rice varieties may result in a varying influence of starch concentration on the hardening rate of rice gels.

The sample temperature prior to homogenizing also strongly influenced gel hardness. The gels prepared from cooked rice maintained at 25°C were softer than those from cooked rice of the same variety maintained at 75°C. Samples of HA1, HA2, and Yume from cooked rice maintained at 75°C showed a higher compressive force than those maintained at 25°C under any concentration throughout storage (Figure 3). This tendency was emphasized by the differences in breaking force. Table 3 compares the breaking forces of gels prepared from rice cooked with 3× water. No fracture point was found for samples on day 0 (all varieties), the HA4 variety under any conditions, and the 25°C sample from Momi on day 1. On days 1 and 3, rice gels from cooked rice maintained at 75°C showed a significantly higher breaking force than those from samples maintained at 25°C for all rice varieties. On day 5, significant differences between 25°C and 75°C were observed for HA1, HA2, Yume, and Momi.

**Table 3:** Comparison of breaking force of rice gels prepared from cooked rice with 3× water^a^

Sample	1 day	3 days	5 days
25°C			
HA1	2.53e	4.02e	5.82d
HA2	7.33b	7.31c	11.92b
HA3	1.83g	2.14h	2.90ef
HA4	–	–	–
Yume	1.90g	1.96h	2.96ef
Momi	–	1.91h	2.56f
Hosh	1.76g	2.01h	2.53f
75°C			
HA1	2.96d	10.43b	10.79c
HA2	8.69a	14.18a	17.67a
HA3	2.99d	3.19f	3.47e
HA4	–	–	–
Yume	3.84c	4.58d	5.45d
Momi	2.09fg	2.65g	3.56e
Hosh	2.38ef	3.16f	2.72f

a In the same column, values with the same letter are not significantly different at *p* < 0.05.

The short‐term development of retrogradation in starch gels is thought to be due to gelation of the solubilized amylose fraction (Miles et al., 1985). Solubilized starch content analysis showed that, for rice cooked with 3× water, the solubilized starch and amylose contents of rice pastes prepared from cooked rice maintained at 25°C were higher than those from the same variety maintained at 75°C. This suggests that factors other than solubilized amylose content contribute to the differences in gel hardness between the 25°C and 75°C samples. The SEM observations demonstrated that the 75°C samples formed a finer gel network following homogenization than the 25°C samples (Figure 1). The textural analysis of cooked rice grains showed that, for almost all samples, the surface firmness of those maintained at 75°C was lower than those maintained at 25°C. The softer texture of the cooked rice surface at 75°C may allow the grains to be homogenized more easily, which may contribute to a finer gel network, resulting in the reinforcement and rigidity of the gel structure. A similar result was reported in the other study (Kokawa et al., 2017), in which the distribution of bubbles in the rice gels contributed to the difference in dynamic viscoelasticity between hot rice gel and cooled rice gel.

Table 4 shows the correlation between starch characteristics (apparent amylose content and amylopectin chain length distribution) and compressive force required to achieve 20% strain in rice gels prepared under various conditions. No significant correlation between gel hardness and apparent amylose content was observed. The peak area ratio of short amylopectin chains (DP6‐12) was negatively correlated with gel hardness for almost all rice gels on days 1, 3, and 5. A positive correlation between gel hardness and the peak area ratio of DP25‐36 chains was observed for rice gels prepared under several cooking conditions. When the weights of water added were compared, rice gels prepared with 3× water had a higher correlation coefficient with the peak area ratio of short amylopectin chains than those prepared with 2× or 4× water. The relationship between the rheological properties of rice starch gels and starch characteristics has been reported in other studies; Takahashi and Fujita (2017) showed using mutant rice varieties that the dynamic viscoelasticity of rice starch gels was strongly affected by the amylose content and the amount of amylopectin short chains. Lii, Lai, and Shen (2004) demonstrated the significant correlation between the dynamic viscoelasticity of rice starch gels and amylose content, the average chain length of amylose, and the weight ratio of long chains and extra‐long to short chains of amylopectin using three high‐amylose cultivars and five low‐amylose cultivars. Vandeputte, Vermeylen, Geeroms, and Delcour (2003) reported that absolute and free amylose content had an impact on starch gel firmness, but showed no significant relationship between rice starch gel texture and amylopectin chain length distribution using waxy, low‐, intermediate‐, and high‐amylose rice cultivars. These observations suggest that the relationships between the rheological properties of rice starch gels and the chemical characteristics of rice starch may depend on the range of variation in amylose content and amylopectin structure of rice samples. The rice samples used in the present study were limited to high‐amylose rice varieties, and the range of amylose content was narrow compared to those used in previous reports because waxy‐, low‐, and intermediate‐amylose rice varieties could not form gels under the same cooking conditions. The presence of unusually long chains (extra‐long chains (ELCs) or super‐long chains (SLCs)) in the amylopectin of high‐amylose rice starch was observed, and these chains cause an overestimation of the apparent amylose content because of their iodine affinity (Hizukuri, Takeda, Maruta, & Juliano, 1989; Takeda, Hizukuri, & Juliano, 1987). This suggests that the presence of ELCs in rice samples may confuse the relationship between apparent amylose content and rice gel hardness. The process of starch gelation during cooling is partly affected by amylose chain re‐association, which is affected by chain length of amylose as well as amylose content and chain length of amylopectin (Chung & Liu, 2009; Seo et al., 2007). This implies that chain length of amylose has an important role in gel formation. Evaluation of the relationship between molecular structure of amylose and gel texture may be helpful to clarify the effects of starch characteristics on the textural properties of rice gels. The ratio of short amylopectin chains (DP6‐12) was observed to strongly influence the difference in hardness of rice gels between rice varieties, except in fresh gels on day 0. The long‐term changes in starch granules during gel storage are attributed to the recrystallization of the amylopectin fraction (Abd Karim et al., 2000). The proportion of amylopectin with DP6‐9 chains was reported to retard amylopectin retrogradation (Shi & Seib, 1992). The positive relationship between the recrystallization of amylopectin and the stiffness of concentrated starch gels has been observed in previous studies (Bertoft et al., 2016; Keetles et al., 1996a; Sasaki, Kawamata, & Okamoto, 2017; Wang & Wang, 2002). This suggests that the short amylopectin chains (DP6‐12) inhibited the recrystallization of amylopectin, resulting in reduced rigidity of the rice gel network.

**Table 4:** Pearson correlation coefficients between starch characteristics and compressive force required for 20% strain for rice gels prepared under various cooking conditions

	Twofold weight of water				Threefold weight of water				Fourfold weight of water			
	0 day	1 day	3 days	5 days	0 day	1 day	3 days	5 days	0 day	1 day	3 days	5 days
25°C												
Apparent amylose	0.69	0.46	0.30	0.23	0.70	0.01	0.04	0.09	0.40	0.65	0.72	0.35
DP6‐12	0.16	−0.77*	−0.89**	−0.90**	0.34	−0.93**	−0.96**	−0.98**	0.43	0.53	−0.31	−0.83*
DP13‐24	0.12	0.71	0.49	0.52	0.21	0.03	0.10	0.22	−0.17	0.11	0.36	0.22
DP25‐36	−0.03	0.50	0.67	0.62	−0.22	0.93**	0.90**	0.88**	−0.12	−0.47	0.31	0.75
75°C												
Apparent amylose	0.62	0.23	0.21	0.26	0.72	0.01	0.16	0.16	0.53	0.19	0.25	0.06
DP6‐12	−0.02	−0.94**	−0.96**	−0.92**	0.27	−0.92**	−0.97**	−0.97**	0.41	−0.83*	−0.92**	−0.92**
DP13‐24	0.23	0.49	0.40	0.52	0.37	0.00	0.39	0.39	0.24	0.10	0.32	0.04
DP25‐36	0.16	0.74	0.77*	0.68	−0.23	0.93**	0.80*	0.80*	−0.30	0.85*	0.81*	0.90**

DP: degree of polymerization.

*^,^**Indicate significance at 0.01–0.05 and <0.01, respectively.

The estimation of relationship between solubilized starch content and gel hardness showed that solubilized starch and amylose contents were negatively correlated with the compressive force for rice gels on day 0 under most preparation conditions. This result indicated that homogenized pastes prepared from cooked rice with more solubilized starch formed softer gels on day 0. The leached‐out starch, mainly amylose, plays a major role in forming a gel network with the swollen granules embedded in the matrix (Mei‐Lin, Chin‐Fung, & Cheng‐Yi, 1997), and Orford, Ring, Carroll, Miles, and Morris (1987) reported that reducing the amount of solubilized amylose would lead to a softer gel. On the other hand, the relationship between the molecular structure of solubilized starch and cooked rice texture has been reported elsewhere. Li, Fitzgerald, Prakash, Nicholson, and Gilbert (2017) demonstrated that stickiness of cooked rice was correlated with the molecular size of leached amylopectin and the proportions of amylopectin chains with DP ≤ 36 in the leached materials. Patindol, Gu, and Wang (2010) showed that the difference in leaching characteristics between rice cultivars was particularly influenced by amylopectin chain length distribution. The effects of molecular structure of leached‐out starch from homogenized pastes on gel hardness should be investigated further to clarify the mechanism of gel formation.

The relationship between the hardness of cooked rice grains and rice gel hardness was estimated. The surface hardness of cooked rice grains maintained at 75°C was significantly correlated with the compressive force of rice gels prepared from cooked rice maintained at 75°C on day 0 (*r* = 0.88, *p* < 0.01), but no significant correlation was otherwise observed.

## 4 CONCLUSIONS

Rice gels, a new material with an expected potential to modify the texture of rice products, were prepared from seven high‐amylose rice varieties by homogenizing cooked rice grains. The starch characteristics of each rice variety were reflected in the differences in rice gel hardness. The proportion of short amylopectin chains (DP6‐12) was found to be negatively correlated with the hardness of rice gels on days 1, 3, and 5 under various preparation conditions. The rice sample temperature before homogenization significantly influenced the compressive force and breaking force of rice gels. The rice gels from cooked rice maintained at 75°C prior to homogenization showed a higher compressive force and breaking force than those from cooked rice maintained at 25°C for almost all of samples. Observations using SEM showed that the cooked rice maintained at 75°C formed a more fibrous and denser structure following homogenization than that maintained at 25°C. The surface of cooked rice grains maintained at 75°C was softer than those maintained at 25°C for almost all. This implies that the softer texture of the cooked rice surface at 75°C may allow the grains to be homogenized more easily, which may contribute to a finer gel network, resulting in the reinforcement and rigidity of the gel structure. These results suggest that the gel network formed in the process of high‐speed shear homogenization had a considerable impact on gel hardness compared to rice flour gel and the optimal combination of rice cultivar, water content, and sample temperature could form rice gels with a wide range of hardness for a variety of applications.

## CONFLICT OF INTEREST

The authors declare that they do not have any conflict of interest.

## ETHICAL REVIEW

This study does not involve any human or animal testing.
